# Sex disparities in the prevalence, incidence, and management of diabetes mellitus: an Australian retrospective primary healthcare study involving 668,891 individuals

**DOI:** 10.1186/s12916-024-03698-0

**Published:** 2024-10-16

**Authors:** George Mnatzaganian, Crystal Man Ying Lee, Gill Cowen, James H. Boyd, Richard J. Varhol, Sean Randall, Suzanne Robinson

**Affiliations:** 1 La Trobe Rural Health School, College of Science, Health, and Engineering, La Trobe University, Bendigo, VIC Australia; 2 School of Population Health, Curtin University, Perth, WA Australia; 3 Curtin Medical School, Curtin University, Bentley, WA Australia; 4 Curtin Health Innovation Research Institute (CHIRI), Curtin University, Bentley, WA Australia; 5 Department of Public Health, La Trobe University, Bundoora, VIC Australia; 6 Deakin Health Economics, School of Health and Social Development, Institute for Health Transformation, Faculty of Health Deakin University, Geelong, VIC Australia

**Keywords:** Diabetes, Sex, Prevalence, Incidence, Health targets

## Abstract

**Background:**

In Australia, diabetes is the fastest growing chronic condition, with prevalence trebling over the past three decades. Despite reported sex differences in diabetes outcomes, disparities in management and health targets remain unclear. This population-based retrospective study used MedicineInsight primary healthcare data to investigate sex differences in diabetes prevalence, incidence, management, and achievement of health targets.

**Methods:**

Adults (aged ≥ 18 years) attending 39 general practices in Western Australia were included. Diabetes incidence and prevalence were estimated by age category. Health targets assessed included body mass index (BMI), blood pressure, blood lipids, and glycated haemoglobin (HbA_1c_) levels. Medical management of diabetes-associated conditions was also investigated. Time-to-incident diabetes was modelled using a Weibull regression. A multilevel mixed-effects logistic regression model investigated risk-adjusted sex differences in achieving the HbA_1c_ health target (HbA_1c_ ≤ 7.0% (≤ 53 mmol/mol)).

**Results:**

Records of 668,891 individuals (53.4% women) were analysed. Diabetes prevalence ranged from 1.3% (95% confidence interval (CI) 1.2%-1.3%) in those aged < 50 years to 7.2% (95% CI 7.1%-7.3%) in those aged ≥ 50 years and was overall higher in men. In patients younger than 30 years, incidence was higher in women, with this reversing after the age of 50. Among patients with diabetes, BMI ≥ 35 kg/m^2^ was more prevalent in women, whereas current and past smoking were more common in men. Women were less likely than men to achieve lipid health targets and less likely to receive prescriptions for lipid, blood pressure, or glucose-lowering agents. Men with incident diabetes were 21% less likely than women to meet the HbA1_c_ target. Similarly, ever recorded retinopathy, nephropathy, neuropathy, hypertension, dyslipidaemia, coronary heart disease, heart failure, peripheral vascular disease and peripheral artery disease were higher in men than women.

**Conclusions:**

This research underscores variations in diabetes epidemiology and management based on sex. Tailoring diabetes management should consider the patient's sex.

**Supplementary Information:**

The online version contains supplementary material available at 10.1186/s12916-024-03698-0.

## Background

Diabetes is a leading cause of disability and mortality worldwide with increasing global prevalence and over 1.5 million deaths directly attributable to it each year [1]. In 2021, the International Diabetes Federation estimated that 537 million people lived with diabetes globally [2], with estimates projected to rise to 1.3 billion in 2050 [3]. Diabetes manifests primarily in two prevalent forms, namely type 1 and type 2. Among these, type 2 diabetes is the more widespread type, responsible for approximately 95% of disability-adjusted life years attributed to this chronic condition [3]. Diabetes is also a key risk factor for stroke and coronary heart disease [4, 5], which are the two global leading causes of disease burden [6].

Previous research has highlighted sex differences in diabetes prevalence, incidence, and outcomes. The global age-standardised diabetes prevalence is higher in men than in women, with a male-to-female ratio of 1.14 (6.5% versus 5.8% respectively) [3]. Compared to women, men are often diagnosed with type 2 diabetes at a younger age and with a lower body mass index (BMI) [7, 8]. In contrast, at the time of diagnosis, women, especially young women, often exhibit a greater burden of risk factors such as higher blood pressure and more obesity than men [9]. Sex differences in diabetes associated outcomes have also been reported with some being worse in women. A systematic review showed that relative risks of developing coronary heart disease and stroke due to diabetes were higher in women compared to men, and after adjusting for major vascular risk factors, diabetes was linked to a nearly 50% higher rate of occlusive vascular mortality among women compared to men [10, 11]. Increased cardiovascular risk factors in women with diabetes and disparities in diabetes treatment favouring men have been suggested as contributing factors [12, 13]. It has been reported that women with diabetes are less likely than their male counterparts to achieve glycaemic control and target levels of glycated haemoglobin (HbA_1c_) [14]. A study conducted in the US found poorer control of blood pressure and low-density lipoprotein (LDL) cholesterol in women compared to men, suggesting that such treatment disparities contributed to the observed sex differences in cardiovascular mortality, to the detriment of women [15].

In Australia, diabetes is the fastest growing chronic condition, increasing at a faster rate than other chronic diseases such as heart disease and cancer, with prevalence trebling over the past three decades [16, 17]. As of 2021, approximately 1 in every 20 Australians was living with diabetes. While there has been a decline in age-standardised diabetes-related mortality over the years, peaking in 2008 (62 per 100,000 population) and steadily declining to 54 per 100,000 population in 2020 [16], there has been an increase in the incidence of medical complications, particularly among men [18]. Despite the increasing prevalence and the sex disparities in diabetes outcomes, it is not known if there are disparities in management and in achievement of health targets in Australia.

To shed more light on this matter, this population-based study investigated sex differences in the prevalence, incidence, and management of diabetes, using a large sample of routinely collected primary healthcare data in Western Australia. This study aimed to provide a sex-stratified snapshot of glycaemic control and diabetes management over the last year of clinical interactions between patients and their general practitioners (GPs). Our approach of examining diabetes management as a snapshot is well-established and frequently employed [19, 20].

## Methods

### Data source and study sample

**Fig. 1:**
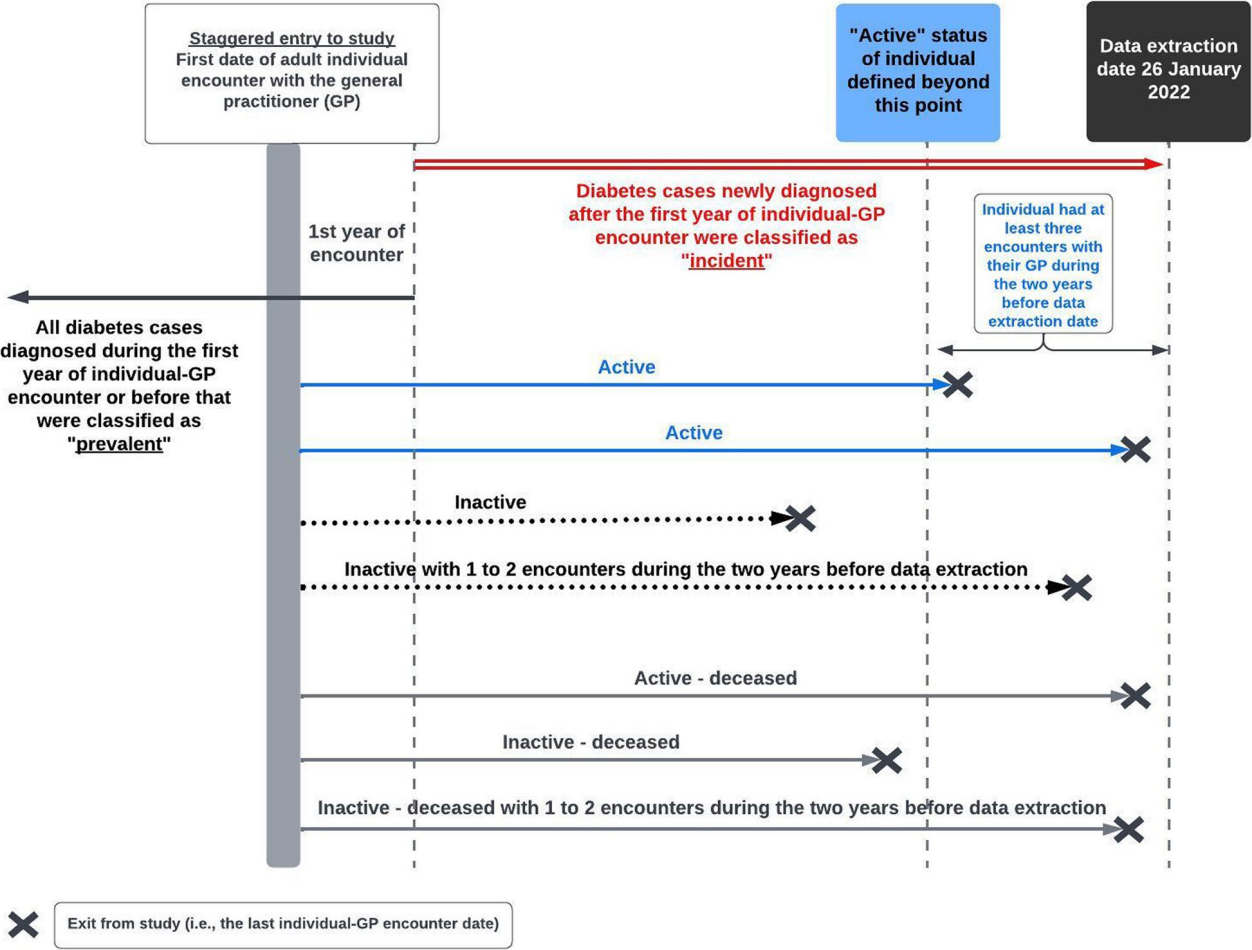
Staggered entry sequence diagram

The study followed a retrospective cohort design with staggered entry. Adults (aged ≥ 18 years) who visited a GP for any reason at one of the 39 MedicineInsight participating general practices in Western Australia were included in this study. As of the data extraction date of January 26, 2022, patients attending these clinics were categorised as follows: "active," defined as having had at least three encounters with the GP in the two years preceding data extraction; "inactive," defined as having had fewer than three such encounters during that period; or "deceased" (Fig. 1).

The de-identified electronic health records were extracted from the MedicineInsight database, an Australian national general practice data programme established by NPS MedicineWise, which included records from general practices that had consented to be part of the programme [21, 22]. The programme uses validated diagnostic algorithms to identify individuals with chronic diseases [23]. Approval for access to the data was given by the NPS MedicineWise Data Governance Committee (2020–003).

Information from MedicineInsight used in this study included demographics, diagnoses, reasons for consultations, laboratory and pathology investigation requests and their results, prescription data, patient screening, anthropometric measurements based on measured weight and height, smoking status and clinical measurements. Dates of diagnoses, tests, referrals, and medical treatments were also available. Socioeconomic disadvantage measure was based on the Socio-Economic Indexes for Areas – Index of Relative Socio-Economic Disadvantage (SEIFA-IRSD) [24], which is a residential postcode-based composite score that ranks geographic areas across Australia according to their relative socioeconomic advantage or disadvantage. All diagnoses were obtained from the “diagnosis”, “reason for encounter” and “reason for prescription” data fields using data extraction methods used by MedicineInsight, including standard clinical terminologies, misspellings, and abbreviations [21–23].

### Ascertainment of diabetes mellitus (type 1, type 2, or unspecified type)

Diabetes mellitus (in this paper, referred to as “diabetes”) case identification was based on recorded diagnoses, prescription reasons, pathology results [25–27], and Medicare Benefits Schedule (MBS) item codes indicating presence of diabetes. MBS is an Australian government-funded list of medical services subsidised for Australian citizens, promoting accessible and affordable healthcare. To optimise the accuracy of diabetes detection, it was necessary to use two distinct records for the same individual to confirm the presence of diabetes [28]. To be defined as having diabetes, a patient needed to meet any of the following criteria:

- 1) Two separate diagnosis records indicating diabetes; 2) two separate HbA_1c_ results ≥6.5% (48 mmol/mol); 3) two separate fasting plasma glucose tests ≥7.0 mmol/L; 4) two separate plasma glucose tests ≥11.1 mmol/L; 5) two separate recorded prescriptions of glucose lowering medications (Anatomical Therapeutic Chemical code: A10); and 6) two separate MBS item codes indicating management or diagnosis of diabetes (codes 66551, 66554, 66841, 73812, 73826, 73839, 73840, 81100, 81105, 81110, 81115, 81120, 81125, and historic codes 2517-2526, and 2620-2635).

The earliest recorded date of any of the above criteria was used as the diagnosis date.

### Exclusion criteria

Without evidence of type 1, type 2, or unspecified type of diabetes, the following conditions were not counted as diabetes for the purposes of this study:

- 1) Gestational diabetes mellitus; 2) pre-diabetes managed with metformin; and 3) polycystic ovary syndrome managed with metformin.

### Definitions

#### Type of diabetes

Individuals identified as having diabetes were categorised as having type 2 if they had a recorded diagnosis indicating type 2 diabetes, non-insulin-dependent diabetes, or adult-onset diabetes. Individuals were recorded as having type 1 if they had a recorded diagnosis indicating type 1 diabetes or insulin-dependent diabetes. Those with a recorded diagnosis of diabetes with an unknown type (for example, “diabetes mellitus”) were classified as having unspecified diabetes. The majority rule was applied in cases where multiple types of diabetes were documented for a patient, determining the patient's classification based on the most frequently documented type. If different types were equally documented, the patient was classified as having "unspecified diabetes".

#### Study entry and exit

Patients entered the study on the initial date of their adult clinical encounter with the GP and exited either upon the patients’ death or upon their last clinical encounter in any of the 39 participating general practices. (Fig. 1).

#### Prevalence versus incidence

Cases diagnosed with diabetes over a period spanning 395 days from the first date of adult clinical encounter or before that were classified as prevalent (Fig. 1). To account for delays in patients’ electronic health recordings, “395 days” instead of the yearly “365 days” was selected. Similarly, patients diagnosed with diabetes based on abnormal HbA_1c_ levels within 12 weeks after 395 days from the first patient-GP encounter were regarded as prevalent cases as HbA_1c_ levels reflect average plasma glucose over the previous 8–12 weeks from the time of the test [29].

Cases diagnosed after 395 days from the first patient-GP encounter (or after 479 days for HbA_1c_ criterion) were classified as incident cases. All prevalent cases were excluded from the incidence estimation. Women who had a history of gestational diabetes but did not show evidence of type 1, type 2, or unspecified diabetes were included among those at risk of developing diabetes mellitus as such women were at high risk of developing type 2 diabetes [30, 31].

Cases with unknown diagnosis date were classified as unknown prevalent or incident diabetes.

#### Patient-GP consultations

Multiple consultations occurring on the same day for the same patient were considered as single consultations.

#### BMI measurements

We used the BMI estimate recorded in the MedicineInsight database. If this estimate was not available, we computed BMI using the measured weight and height of the participants.

#### Health targets and management

The clinical management goals assessed in this study align with guidelines from the Royal Australian College of General Practitioners (RACGP) [27]. Individual management goals encompass smoking cessation and BMI, while treatment management goals include HbA1_c_, lipid levels, urine albumin creatinine ratio, vaccination, and blood pressure. Screening for potential diabetes-related conditions (ever recorded in patient health records) and the pharmacological approaches to managing blood pressure, dyslipidaemia, and diabetes were also investigated.

### Statistical analysis

Diabetes incidence (all types) (measured as cases per 1,000 person-years of follow-up) and prevalence rates (measured as cases diagnosed over a period spanning 395 days from the first date of adult clinical encounter or before, divided by the number of people in the sample) were estimated with their 95% confidence intervals (CI). Characteristics of the overall cohort as well as individuals with and without diabetes were summarised using standard measures of central tendency and dispersion. Pearson’s χ^2^ test compared the frequencies in categorical variables, while a Mann–Whitney test compared the mean ranks of continuous variables. Prevalence and incidence of diabetes were each compared by the sexes stratified by age groups. Comorbidities and medical conditions were compared by sex stratified by diabetes type and duration.

Clinical and screening measures and health targets were compared by sex and type of diabetes. To be included in this analysis, patients with prevalent diabetes needed at least three years of follow-up while those with incident diabetes required three years of follow-up after their diabetes diagnosis.

### Multivariable analysis: time to incident diabetes

The proportional hazards assumption was violated, rendering Cox regression unsuitable for analysis. Instead, time-to-incident diagnosis of diabetes (all types) was modelled using an accelerated time Weibull regression which provided the best fit with the lowest Akaike Information Criteria (AIC) compared to other parametric survival distributions. Study participants without evidence of prevalent diabetes were followed up from the first adult clinical encounter until they were diagnosed with incident diabetes or died or were right censored at the last clinical encounter. The analysis adjusted for age at first clinical encounter, sex, SEIFA-IRSD, smoking status and BMI (both at the first adult clinical encounter), and Indigenous ethnicity, while also accounting for clustering effects within the 39 participating general practices. Risk adjusted probability of remaining free of diabetes over time was plotted by sex.

### Multivariable analysis: achievement of glycaemic control in the last year of clinical encounter among patients with incident diabetes

Sex differences in achieving glycaemic control [HbA1_c_ ≤ 7.0% (≤ 53 mmol/mol)] (yes/no) over a period of up to 395 days, ending at the last clinical encounter, in patients with incident type 2 or unspecified diabetes who had at least three years of follow-up from diagnosis, were modelled using a multilevel mixed effects logistic regression. The multivariable model adjusted for 1) demographics (sex, age at last clinical encounter, SEIFA-IRSD, and Indigenous ethnicity); 2) BMI and smoking status as recorded over a period spanning 395 days up to the last clinical encounter); 3) years of follow-up; 4) active status of the patient; 5) baseline adult first recorded HbA1_c_ level; and 5) clinical conditions that could have resulted in falsely high or falsely low HbA1_c_ levels including anaemia, chronic kidney disease, chronic liver disease, hypertriglyceridaemia, and pregnancy [32–35]. The model also adjusted for cluster effects within the 39 participating general practices.

### Multivariable analysis: sex differences in diabetes management in prevalent and incident cases

Sex disparities in the absence of tests or clinical assessments, lack of screening for diabetes-related conditions, and non-treatment with medications for diabetes-associated conditions over a period spanning 395 days up to the last clinical encounter were each analysed using multilevel mixed-effects logistic regression models. These models were adjusted for age at the last clinical encounter, sex, SEIFA-IRSD, smoking status, BMI, Indigenous ethnicity, rurality, duration of follow-up, and type of diabetes. Additionally, the models accounted for clustering effects within the 39 participating general practices.

The analyses were conducted separately for prevalent and incident cases, each requiring at least three years of follow-up from their initial adult clinical encounter or from their diabetes incident diagnosis, respectively.

### Sensitivity analyses

In sensitivity analyses, sex differences in achieving glycaemic control were separately modelled after excluding pregnant women (over the period of 395 days up to the last clinical encounter) and/or after limiting the analyses to type 2 diabetes.

All analyses were performed using Stata/MP 17.0 (StataCorp, College Station, TX, USA).

## Results

**Fig. 2:**
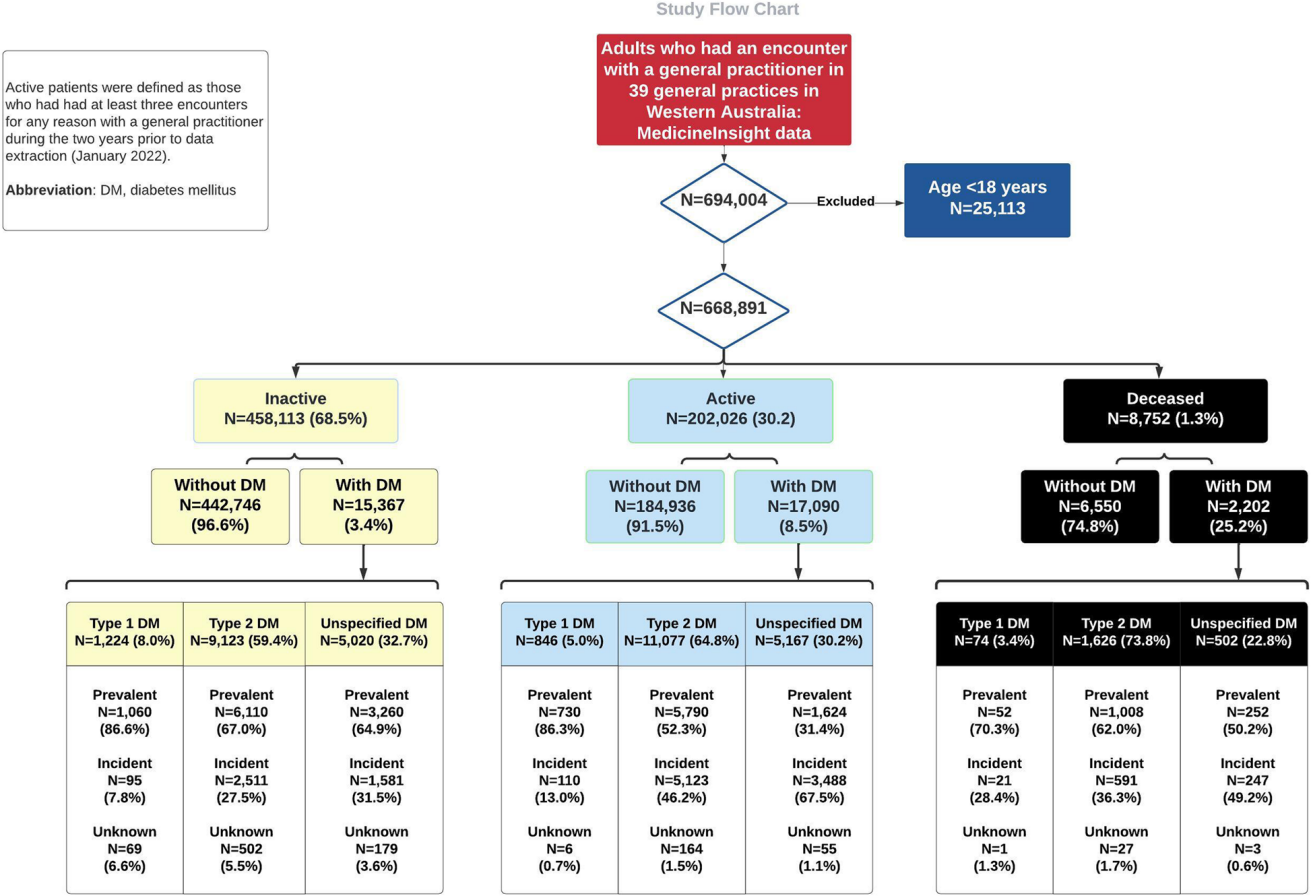
Cohort selection – study flow chart

Records of 668,891 individuals (53.4% women, mean follow-up 3.7 ± 5.1 years) from 39 general practices in Western Australia were analysed. Of these, 202,026 (30.2%) were classified as “active”, 458,113 (68.5%) as “inactive”, and 8,752 (1.3%) as “deceased” at the time of data extraction. Within these categories, diabetes was identified in 8.5%, 3.4%, and 25.2%, respectively. Among the total 34,659 patients diagnosed with diabetes, 6.2% were classified as type 1 diabetes, 63.0% as type 2 diabetes, and 30.8% had unspecified diabetes (Fig. 2). Compared to those without diabetes, patients with diabetes were more likely to be older, male, overweight or obese, to come from disadvantaged socioeconomic backgrounds, and to have had more consultations over a period of 395 days up to the last year of clinical encounter with longer years of follow-up (7.3 ± 6.5 years in those with diabetes versus 3.5 ± 6.5 years in those without) (Additional file 1: Table S1).

As expected, type 1 diabetes was predominantly (85.9%) prevalent in our adult cohort, whereas 37.7% of type 2 diabetes and 49.7% of unspecified type cases were incident diabetes (Fig. 2).

**Fig. 3:**
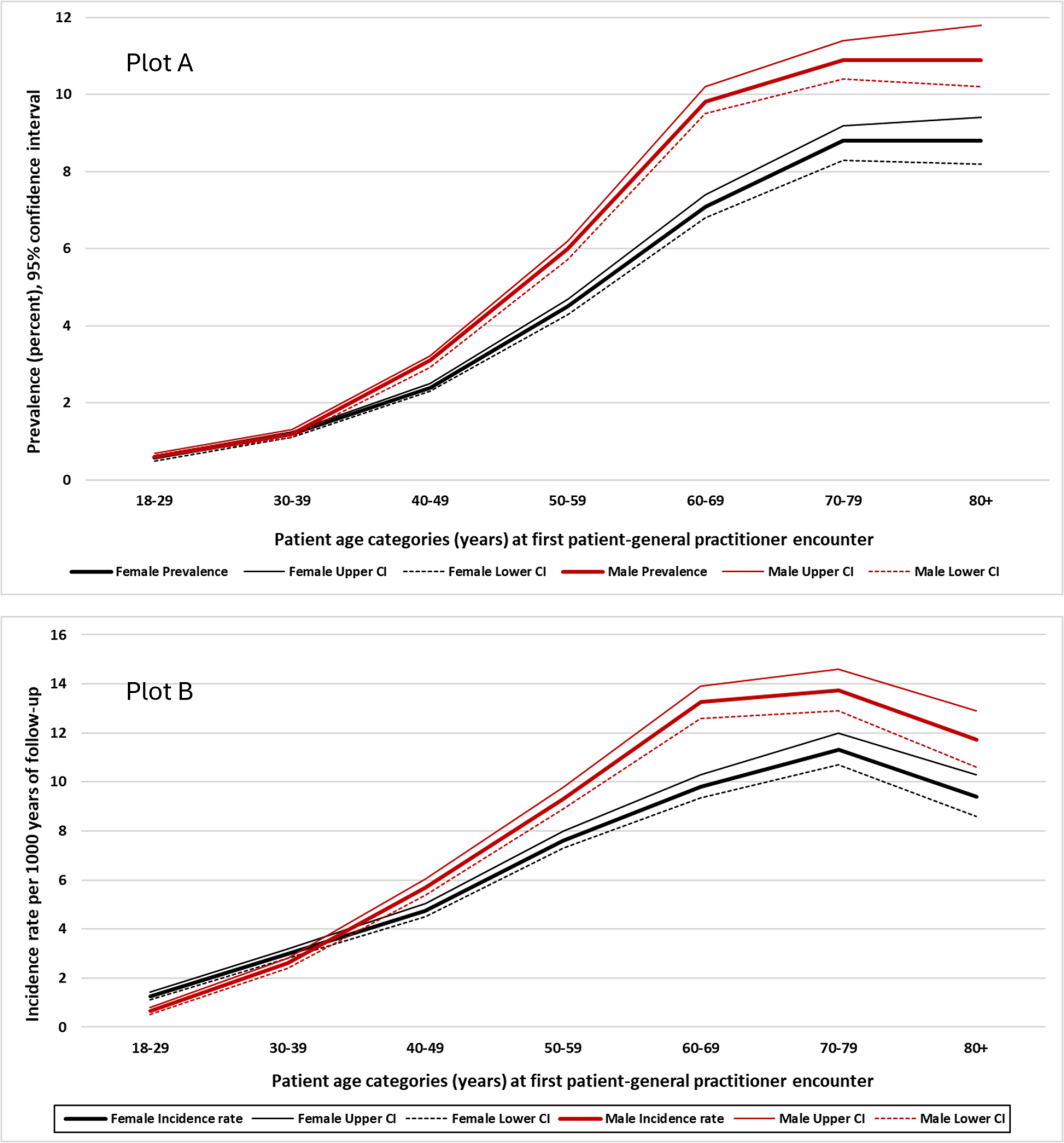
Prevalence and incidence of diabetes mellitus by sex and age category

Prevalence and incidence estimates of diabetes (all types) classified by the “active” patient status, age and sex are presented in Table 1. Overall, the prevalence among those aged ≥ 50 years was 7.2% (95% CI 7.1%—7.3%), being significantly higher in men (8.3% (95% CI 8.1% – 8.5%)) than in women (6.3% (95% CI 6.2%—6.5%)), *p* < 0.001. The prevalence was also higher in men among individuals younger than 50 years old (Fig. 3, Plot A).

**Table 1:** Prevalence and incidence rates of diabetes (all types) by sex, age category, and active status^a^ of the individual

		Women *N* = 356,910		Men *N* = 305,719		All^b^ *N* = 668,891	
		Age <50 years	Age ≥50 years	Age <50 years	Age ≥50 years	Age <50 years	Age ≥50 years
Prevalence Percent (95% CI)	**Active**	1.7 (1.6 – 1.7)	7.6 (7.3 – 7.8)	2.1 (2.0 – 2.3)	10.1 (9.7 – 10.4)	1.9 (1.8 – 1.9)	8.7 (8.5 – 8.9)
	**Inactive**	0.9 (0.8 – 1.0)	5.2 (5.1 – 5.4)	1.1 (1.0 – 1.2)	6.7 (6.4 – 6.9)	1.0 (0.9 – 1.0)	5.8 (5.7 – 6.0)
	**Deceased**	8.9 (6.6 – 12.0)	15.0 (13.8 – 16.2)	6.5 (4.9 – 8.7)	17.0 (15.9 – 18.2)	7.5 (6.1 – 9.2)	16.1 (15.2 – 16.9)
	**All**	1.2 (1.1 – 1.2)	6.3 (6.2 – 6.5)	1.4 (1.3 – 1.5)	8.3 (8.1 – 8.5)	1.3 (1.2 – 1.3)	7.2 (7.1 – 7.3)
Incidence rate per 1000 years (95% CI)	**Active**	5.7 (5.5 – 6.0)	10.5 (10.0 – 11.0)	5.9 (5.7 – 6.2)	13.1 (12.6 – 13.7)	5.8 (5.7 – 6.0)	11.7 (11.3 – 12.1)
	**Inactive**	1.9 (1.8 – 2.0)	7.5 (7.1 – 8.0)	2.5 (2.4 – 2.7)	9.8 (9.2 – 10.4)	2.2 (2.1 – 2.3)	8.5 (8.2 – 8.9)
	**Deceased**	11.9 (8.8 – 16.1)	13.7 (12.3 – 15.3)	10.8 (8.2 – 14.1)	16.4 (15.0 – 18.1)	11.2 (9.2 – 13.7)	15.1 (14.1 – 16.2)
	**All**	3.8 (3.7 – 3.9)	9.5 (9.2 – 9.8)	4.2 (4.0 – 4.4)	12.1 (11.7 – 12.5)	4.0 (3.9 – 4.1)	10.7 (10.4 – 10.9)

^a^Active status was defined as having at least three encounters for any reason with a general practitioner during the two years prior to data extraction date (January 2022)

^b^The total sample also included 6,262 individuals whose sex was either different from male or female or was unknown

**Fig. 4:**
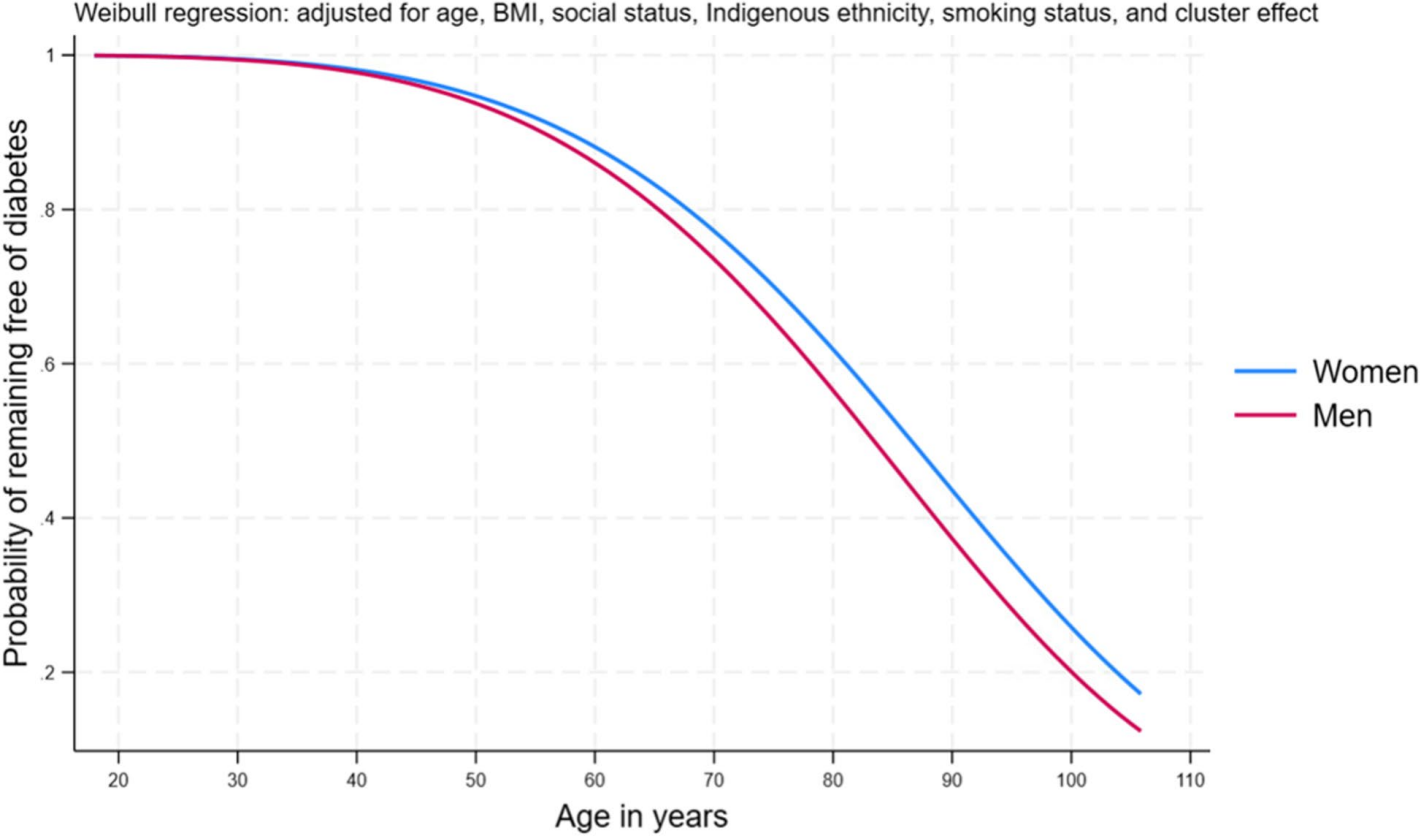
Risk-adjusted probability of remaining free of diabetes by sex

The overall incidence rate of diabetes per 1000 years of follow-up was 5.9 (95% CI 5.8–6.0), ranging from 4.0 per 1000 years (95% CI 3.9–4.1) among those younger than 50 years to 10.7 per 1000 years (95% CI 10.4–10.9) in those aged ≥ 50 years (Table 1). In patients ≤ 30 years, diabetes incidence was higher in women compared to men; however, higher incident rates in men were consistently observed after the age of 50 years (Fig. 3, Plot B and Fig. 4).

Adjusting for age, BMI and smoking status (all three as recorded at first adult clinical encounter), and Indigenous ethnicity, socioeconomic disadvantage, and cluster effect within the general practices, men were 19% more likely than women to be diagnosed with incident diabetes (adjusted hazard ratio 1.19, 95% CI 1.09–1.30, *p* < 0.001) (Additional file 1: Table S2). Over time, the increased risk was considerably higher in men than women as shown in Fig. 4.

At the last clinical encounter, burden of diseases varied by the sexes who had diabetes. Ever recorded coronary heart disease, heart failure, hypertension, peripheral vascular disease and peripheral artery disease were significantly higher in men compared to women (Table 2). In type 1 diabetes, diabetes associated metabolic conditions, specifically hypoglycaemia, were more common in women. Stroke and transient ischaemic attack were evenly distributed in both men and women with type 1 and type 2 diabetes; however, these were more commonly reported in men with unspecified diabetes type. In all types of diabetes combined, the prevalence of most of the conditions was higher in men than in women (Additional file 1: Table S3). Years of follow-up in all types of diabetes were slightly higher in women compared to men (mean ± standard deviation 7.4 ± 6.6 years versus 7.2 ± 6.5 years).

**Table 2:** Ever recorded conditions by type, duration of diabetes mellitus, and sex^a^ at the last clinical encounter: n (%)

	Type 1 diabetes mellitus				Type 2 diabetes mellitus				Unspecified diabetes mellitus			
	Diabetes duration 1 to 10 years		Diabetes duration >10 years		Diabetes duration 1 to 10 years		Diabetes duration >10 years		Diabetes duration 1 to 10 years		Diabetes duration >10 years	
	Women	Men	Women	Men	Women	Men	Women	Men	Women	Men	Women	Men
	*N*=480	*N*=603	*N*=468	N=512	*N*=5829	*N*=6858	*N*=3922	*N*=4472	*N*=4769	*N*=3660	*N*=1027	*N*=957
Retinopathy	13 (2.7)	19 (3.2)	47 (10.0)	68 (13.3)	89 (1.5)	119 (1.7)	175 (4.5)	238 (5.3)	21 (0.4)	21 (0.6)	22 (2.1)	30 (3.1)
Nephropathy	18 (3.8)	19 (3.2)	23 (4.9)	37 (7.2)	404 (6.9)	528 (7.7)	498 (12.7)	591 (13.2)	119 (2.5)	137 (3.7)^**^	76 (7.4)	77 (8.1)
Neuropathy	23 (4.8)	29 (4.8)	36 (7.7)	47 (9.2)	449 (7.7)	565 (8.2)	409 (10.4)	551 (12.3)^*^	260 (5.4)	185 (5.1)	61 (5.9)	64 (7.7)
Hypertension	57 (11.9)	117 (19.4)^**^	120 (25.6)	156 (30.5)	3194 (54.8)	3784 (55.2)	2716 (69.2)	2954 (66.1)^*^	1399 (29.3)	1361 (37.2)^**^	499 (48.6)	454 (47.4)
Dyslipidaemia	53 (11.0)	87 (14.4)	102 (21.8)	105 (20.5)	2459 (42.2)	2955 (43.1)	2022 (51.6)	2229 (49.8)	1117 (23.4)	1006 (27.5)^**^	361 (35.2)	305 (31.9)
PVD / PAD	4 (0.8)	5 (0.8)	12 (2.6)	20 (3.9)	83 (1.4)	191 (2.8)^**^	145 (3.7)	278 (6.2)^**^	21 (0.4)	49 (1.3)^**^	20 (1.9)	26 (2.7)
CHD	22 (4.6)	32 (5.3)	51 (10.9)	54 (10.6)	695 (11.9)	1292 (18.8)^**^	850 (21.7)	1351 (30.2)^**^	281 (5.9)	456 (12.5)^**^	159 (15.5)	228 (23.8)^**^
Heart failure	6 (1.3)	4 (0.7)	16 (3.4)	13 (2.5)	234 (4.0)	340 (5.0)^*^	355 (9.1)	461 (10.3)	93 (2.0)	135 (3.7)^**^	65 (6.3)	77 (8.1)
Stroke/TIA	7 (1.5)	12 (2.0)	20 (4.3)	34 (6.6)	600 (10.3)	648 (9.5)	626 (16.0)	662 (14.8)	290 (6.1)	247 (6.7)	86 (8.4)	107 (11.2)^*^
Cancer	28 (5.8)	30 (5.0)	58 (12.4)	51 (10.0)	1148 (19.7)	1236 (18.0)^*^	1107 (28.2)	1380 (30.9)^*^	641 (13.4)	495 (13.5)	204 (19.9)	186 (19.4)
Metabolic^b^	25 (5.2)	16 (2.7)^*^	40 (8.6)	21 (4.1)^*^	26 (0.5)	31 (0.5)	33 (0.8)	33 (0.7)	14 (0.3)	6 (0.2)	9 (0.9)	4 (0.4)
Other^c^	1 (0.2)	2 (0.3)	2 (0.4)	3 (0.6)	35 (0.6)	23 (0.3)^*^	29 (0.7)	24 (0.5)	23 (0.5)	12 (0.3)	3 (0.3)	4 (0.4)

*Abbreviations*: *CHD *Coronary heart disease, *PAD *Peripheral artery disease, *PVD *Peripheral vascular disease, *TIA *Transient ischaemic attack

Sexes were compared in each diabetes duration period in each type of diabetes with ≤0.05 * ≥0.001; **<0.001

^a^Not included in the table: 108 individuals with unknown sex, and 524 and 470 males and females, respectively, who did not have a known diabetes diagnosis date

^b^Metabolic conditions included diabetic ketoacidosis (DKA), hyperglycaemic non-ketotic coma (HONK), and hypoglycaemia

cOther included cheiroarthropathy and periodontitis

Sex differences were consistently observed in diabetes management over the period spanning 395 days up to the last clinical encounter, whether for prevalent cases (Table 3) or newly diagnosed ones (Table 4). Approximately 77.4% and 86.7% of prevalent and incident cases, respectively were overweight or obese, with obesity class II and class III or more being significantly more prevalent among women, *p* < 0.001. BMI ≥ 35 kg/m^2^ was significantly less prevalent in patients with type 1 diabetes compared to those with type 2 or unspecified diabetes, consistently observed in both sexes (Tables 3 and 4). Women were less likely than men to achieve lipid health targets and less likely to be prescribed with lipid modifying agents over the period of 395 days up to the last clinical encounter (35.7% in women versus 44.7% in men, *p* < 0.001). Sex differences in lipid management remained after limiting this comparison to those with a confirmed diagnosis of dyslipidaemia (62.3% in women versus 69.0% in men, *p* < 0.001). Confined to those with a confirmed diagnosis of hypertension, management with blood pressure lowering agents over the period of 395 days up to the last clinical encounter was also significantly lower in women (74.3%) than in men (76.2%), *p* = 0.003. Similarly, women diagnosed with diabetes were significantly less likely than their male counterparts to receive glucose lowering medications, (56.0% in women versus 59.2% in men, *p* < 0.001). Sex disparities in lipid, blood pressure, and glucose management remained consistent when the analyses were stratified by prevalent or incident diabetes among patients with at least three years of follow-up as shown in Tables 3 and 4.

**Table 3:** Sex-stratified age, follow-up, health targets, clinical management goals, prescription, and screening over a period spanning 395 days up to the last clinical encounter in patients with a prevalent diabetes with at least 3 years of follow-up: n (%) if not otherwise stated

	All types of DM		Type 1 DM		Type 2 DM		Unspecified type DM	
	Women *N*=4,580	Men *N*=5,078	Women *N*=417	Men *N*=493	Women *N*=3,385	Men *N*=3,875	Women *N*=778	Men *N*=710
**Age at last encounter** (years), mean (SD)	65.5 (16.9)	65.3 (15.2)	46.9 (18.3)	47.3 (16.9)	68.6 (14.8)	67.8 (13.1)^*^	61.9 (17.7)	64.2 (15.7)^*^
**Adult follow-up** (years), mean (SD)	8.6 (4.9)	8.2 (4.6)^**^	8.9 (5.3)	8.4 (4.8)	8.8 (5.0)	8.4 (4.7)^**^	7.4 (4.2)	7.4 (4.2)
**HbA1c** ≤7.0% (≤53 mmol/mol)								
Yes	1,496 (32.7)	1,765 (34.8)^**^	64 (15.3)	76 (15.4)	1,261 (37.2)	1,496 (38.6)^**^	171 (22.0)	193 (27.2)^*^
No	1,293 (28.2)	1,613 (31.8)	167 (40.0)	193 (39.1)	995 (29.4)	1,269 (32.7)	131 (16.8)	151 (21.3)
Nottested	1,791 (39.1)	1,700 (33.5)	186 (44.6)	224 (45.4)	1,129 (33.3)	1,110 (28.6)	476 (61.2)	366 (51.5)
**BP** ≤140/90 mm Hg								
Yes	1,331 (29.1)	1,567 (30.9)^**^	158 (37.9)	139 (28.2)^**^	989 (29.2)	1,233 (31.8)^**^	184 (23.6)	195 (27.5)^**^
No	1,760 (38.4)	2,174 (42.8)	82 (19.7)	161 (32.7)	1,481 (43.7)	1,780 (45.9)	197 (25.3)	233 (32.8)
Notmeasured	1,489 (32.5)	1,337 (26.3)	177 (42.4)	193 (39.1)	915 (27.0)	862 (22.2)	397 (51.0)	282 (39.7)
**BP** ≤130/80 mm Hg								
Yes	546 (11.9)	625 (12.3)^**^	80 (19.2)	66 (13.4)^*^	380 (11.2)	477 (12.3)^**^	86 (11.0)	82 (11.5)^**^
No	2,545 (55.6)	3,116 (61.4)	160 (38.4)	234 (47.5)	2,090 (61.7)	2,536 (65.4)	295 (37.9)	346 (48.7)
Not measured	1,489 (32.5)	1,337 (26.3)	177 (42.4)	193 (39.1)	915 (27.0)	862 (22.2)	397 (51.0)	282 (39.7)
**Total cholesterol** <4.0 mmol/L								
Yes	697 (15.2)	1,293 (25.5)^**^	39 (9.3)	58 (11.8)	581 (17.2)	1,136 (29.3)^**^	77 (9.9)	99 (13.9)^*^
No	1,689 (36.9)	1,655 (32.6)	141 (33.8)	158 (32.0)	1,354 (40.0)	1,298 (33.5)	194 (24.9)	199 (28.0)
Not tested	2,194 (47.9)	2,130 (41.9)	237 (56.8)	277 (56.2)	1,450 (42.8)	1,441 (37.2)	507 (65.2)	412 (58.0)
**LDL-C**^a^								
Yes	767 (16.7)	1,140 (22.4)^**^	36 (8.6)	37 (7.5)	651 (19.2)	1,013 (26.1)^**^	80 (10.3)	90 (12.7)^*^
No	1,425 (31.1)	1,531 (30.2)	124 (29.7)	154 (31.2)	1,134 (33.5)	1,194 (30.8)	167 (21.5)	183 (25.8)
Nottested	2,388 (52.1)	2,407 (47.4)	257 (61.6)	302 (61.3)	1,600 (47.3)	1,668 (43.0)	531 (68.2)	437 (61.5)
**HDL-C** ≥1.0 mmol/L								
Yes	1,930 (42.1)	1,793 (35.3)^**^	152 (36.4)	174 (35.3)	1,545 (45.6)	1,425 (36.8)^**^	233 (29.9)	194 (27.3)^**^
No	326 (7.1)	984 (19.4)	10 (2.4)	23 (4.7)	291 (8.6)	877 (22.6)	25 (3.2)	84 (11.8)
Nottested	2,324 (50.7)	2,301 (45.3)	255 (61.1)	296 (60.0)	1,549 (45.8)	1,573 (40.6)	520 (66.8)	432 (60.8)
**Triglycerides** <2.0 mmol/L								
Yes	1,348 (29.4)	1,631 (32.1)^**^	156 (37.4)	167 (33.9)^*^	1,018 (30.1)	1,303 (33.6)^**^	174 (22.4)	161 (22.7)^*^
No	1,028 (22.4)	1,309 (25.8)	23 (5.5)	48 (9.7)	910 (26.9)	1,125 (29.0)	95 (12.2)	136 (19.1)
Nottested	2,204 (48.1)	2,138 (42.1)	238 (57.1)	278 (56.4)	1,457 (43.0)	1,447 (37.3)	509 (65.4)	413 (58.2)
**Non-HDL-C** <2.5 mmol/L								
Yes	80 (1.7)	153 (3.0)^**^	4 (1.0)	8 (1.6)	67 (2.0)	135 (3.5)^**^	9 (1.2)	10 (1.4)
No	263 (5.7)	290 (5.7)	22 (5.3)	30 (6.1)	202 (6.0)	218 (5.6)	39 (5.0)	42 (5.9)
Not tested	4,237 (92.5)	4,635 (91.3)	391 (93.8)	455 (92.3)	3,116 (92.0)	3,522 (90.9)	730 (93.8)	658 (92.7)
**Urine albumin-creatinine ratio (uACR)**^b^								
Yes	1,285 (28.1)	1,362 (26.8)^**^	113 (27.1)	146 (29.6)	1,034 (30.5)	1,074 (27.7)^**^	138 (17.7)	142 (20.0)^*^
No	665 (14.5)	1,120 (22.1)	38 (9.1)	58 (11.8)	561 (16.6)	974 (25.1)	66 (8.5)	88 (12.4)
Nottested	2,630 (57.4)	2,596 (51.1)	266 (63.8)	289 (58.6)	1,790 (52.9)	1,827 (47.1)	574 (73.8)	480 (67.6)
**Glucose lowering medications** (ever prescription)^I^	4,028 (88.0)	4,545 (89.5)^*^	387 (92.8)	462 (93.7)	3,024 (89.3)	3,519 (90.8)^*^	617 (79.3)	564 (79.4)
**Glucose lowering medications** (prescription over a period of 395 days up to last encounter)^I^	2,856 (62.4)	3,496 (68.8)^**^	270 (64.7)	332 (67.3)	2,269 (67.0)	2,821 (72.8)^**^	317 (40.7)	343 (48.3)^*^
**Lipid modifying agents** (ever prescription)^II^	3,090 (67.5)	3,692 (72.7)^**^	158 (37.9)	202 (41.0)	2,523 (74.5)	3,054 (78.8)^**^	409 (52.6)	436 (61.4)^*^
**Lipid modifying agents** (prescription over a period of 395 days up to last encounter)^II^	2,159 (47.1)	2,791 (55.0)^**^	97 (23.3)	141 (28.6)	1,818 (53.7)	2,367 (61.1)^**^	244 (31.4)	283 (39.9)^*^
**Blood pressure lowering agents** (ever prescription)^III^	3,842 (83.9)	4,217 (83.0)	285 (68.3)	286 (58.0)^*^	2,990 (88.3)	3,396 (87.6)	567 (72.9)	535 (75.3)
**Blood pressure lowering agents** (prescription over a period of 395 days up to last encounter)^III^	2,750 (60.0)	3,227 (63.5)^**^	154 (36.9)	174 (35.3)	2,270 (67.1)	2,695 (69.5)^*^	326 (41.9)	358 (50.4)^*^
**BMI** (kg/m^2^)								
Underweight: <18.5	16 (0.3)	4 (0.1)^**^	7 (1.7)	2 (0.4)^**^	5 (0.1)	1 (0.0)^**^	4 (0.5)	1 (0.1)^**^
Normal weight: 18.5-24.9	360 (7.9)	384 (7.6)	91 (21.8)	89 (18.0)	212 (6.3)	237 (6.1)	57 (7.3)	58 (8.2)
Overweight: 25.0-29.9	854 (18.6)	1,315 (25.9)	77 (18.5)	158 (32.0)	630 (18.6)	981 (25.3)	147 (18.9)	176 (24.8)
Obese class I: 30.0-34.9	995 (21.7)	1,386 (27.3)	88 (21.1)	94 (19.1)	763 (22.5)	1,139 (29.4)	144 (18.5)	153 (21.5)
Obese class II: 35.0-39.9	783 (17.1)	740 (14.6)	44 (10.5)	32 (6.5)	634 (18.7)	605 (15.6)	105 (13.5)	103 (14.5)
Obese class III: ≥40.0	842 (18.4)	561 (11.0)	27 (6.5)	14 (2.8)	699 (20.6)	484 (12.5)	116 (14.9)	63 (8.9)
Notmeasured	730 (15.9)	688 (13.5)	83 (19.9)	104 (21.1)	442 (13.1)	428 (11.0)	205 (26.3)	156 (22.0)
**Smoking**								
Non-smoker	2,094 (45.7)	1,565 (30.8)^**^	182 (43.6)	200 (40.6)	1,642 (48.5)	1,179 (30.4)^**^	270 (34.7)	186 (26.2)^**^
Current	373 (8.1)	536 (10.6)	48 (11.5)	77 (15.6)	269 (7.9)	397 (10.2)	56 (7.2)	62 (8.7)
Past	1,652 (36.1)	2,526 (49.7)	148 (35.5)	170 (34.5)	1,192 (35.2)	1,991 (51.4)	312 (40.1)	365 (51.4)
Notrecorded	461 (10.1)	451 (8.9)	39 (9.3)	46 (9.3)	282 (8.3)	308 (7.9)	140 (18.0)	97 (13.7)
**Influenza vaccination**, ever	2,252 (49.2)	2,448 (48.2)	166 (39.8)	183 (37.1)	1,853 (54.7)	2,053 (53.0)	233 (30.0)	212 (29.9)
**Influenza vaccination**, over a period of 395 days up to last encounter	1,132 (24.7)	1,179 (23.2)	70 (16.8)	71 (14.4)	944 (27.9)	1,016 (26.2)	118 (15.2)	92 (13.0)
**Pneumococcal vaccination**, ever	453 (9.9)	488 (9.6)	24 (5.8)	13 (2.6)^*^	399 (11.8)	445 (11.5)	30 (3.9)	30 (4.2)
**Ophthalmological review**, ever	1,776 (38.8)	1,918 (37.8)	142 (34.0)	142 (28.8)	1,446 (42.7)	1,584 (40.9)	188 (24.2)	192 (27.0)
**Referral to a podiatrist**, ever	710 (15.5)	858 (16.9)	52 (12.5)	71 (14.4)	563 (16.6)	696 (18.0)	95 (12.2)	91 (12.8)

*Abbreviations*: *BMI *Body mass index, *BP *Blood pressure, *DM *Diabetes mellitus, *HbA1c* Glycated haemoglobin, *HDL *High density lipoprotein, *LDL *Low density lipoprotein

^a^LDL_C taret: <2.0 mmol/L or <1.8 mmol/L for those with established CVD (in this analysis these included coronary heart disease, cerebrovascular disease/stroke, or heart failure)

^b^Urine Albumin-creatine ratio (uARC): <3.5 mg/mmol in women and <2.5 mg/mmol in men

^I^ATC code A10; ^II^ ATC code C10; ^III^ ATC codes C02-C04, C07-C09

**Table 4:** Sex-stratified age, follow-up, health targets, clinical management goals, prescription, and screening over a period spanning 395 days up to the last clinical encounter in patients with incident diabetes who have been followed up for at least 3 years following their diabetes diagnosis: n (%) if not otherwise stated

	All types of DM		Type 1 DM		Type 2 DM		Unspecified type DM	
	Women*N*=3,620	Men*N*=3,840	Women*N*=63	Men*N*=68	Women*N*=2,610	Men*N*=2,997	Women*N*=947	Men*N*=775
**Age at last encounter** (years), mean (SD)	66.8 (15.1)	67.3 (13.6)	50.9 (17.4)	54.0 (18.9)	68.4 (12.9)	67.7 (13.0)	63.6 (17.0)	66.5 (14.6)^**^
**Follow-up** (years), mean (SD)	14.4 (5.3)	14.4 (5.3)	12.0 (5.0)	12.5 (5.0)	14.7 (5.3)	14.7 (5.3)	13.8 (5.4)	13.5 (5.2)
**HbA1c** ≤7.0% (≤53 mmol/mol)								
Yes	1,595 (44.1)	1,746 (45.5)^**^	8 (12.7)	13 (19.1)	1,298 (49.7)	1,422 (47.4)^**^	289 (30.5)	311 (40.1)^**^
No	686 (18.9)	975 (25.4)	27 (42.9)	26 (38.2)	579 (22.2)	832 (27.8)	80 (8.5)	117 (15.1)
Nottested	1,339 (37.0)	1,119 (29.1)	28 (44.4)	29 (42.6)	733 (28.1)	743 (24.8)	578 (61.0)	347 (44.8)
**BP** ≤140/90 mm Hg								
Yes	1,066 (29.4)	1,146 (29.8)^**^	20 (31.7)	19 (27.9)	784 (30.0)	908 (30.0)	262 (27.7)	219 (28.3)^**^
No	1,629 (45.0)	1,868 (48.7)	16 (25.4)	21 (30.9)	1,276 (48.9)	1,503 (50.1)	337 (35.6)	344 (44.4)
Notmeasured	925 (25.6)	826 (21.5)	27 (42.9)	28 (41.2)	550 (21.1)	586 (19.6)	348 (36.7)	212 (27.3)
**BP** ≤130/80 mm Hg								
Yes	407 (11.2)	423 (11.0)^**^	9 (14.3)	8 (11.8)	277 (10.6)	332 (11.1)	121 (12.8)	83 (10.7)^**^
No	2,288 (63.2)	2,591 (67.5)	27 (42.9)	32 (47.1)	1,783 (68.3)	2,079 (69.4)	478 (50.5)	480 (61.9)
Not measured	925 (25.6)	826 (21.5)	27 (42.9)	28 (41.2)	550 (21.1)	586 (19.6)	348 (36.7)	212 (27.3)
**Total cholesterol** <4.0 mmol/L								
Yes	504 (13.9)	1,019 (26.5)^**^	4 (6.4)	9 (13.2)	434 (16.6)	861 (28.7)^**^	66 (7.0)	149 (19.2)^**^
No	1,588 (43.9)	1,469 (38.3)	27 (42.9)	24 (35.3)	1,225 (46.9)	1,173 (39.1)	336 (35.5)	272 (35.1)
Not tested	1,528 (42.2)	1,352 (35.2)	32 (50.8)	35 (51.5)	951 (36.4)	963 (32.1)	545 (57.5)	354 (45.7)
**LDL-C**^a^								
Yes	576 (15.9)	889 (23.1)^**^	4 (6.3)	9 (13.2)	498 (19.1)	750 (25.0)^**^	74 (7.8)	130 (16.8)^**^
No	1,328 (36.7)	1,371 (35.7)	23 (36.5)	21 (30.9)	1,005 (38.5)	1,104 (36.8)	300 (31.7)	246 (31.7)
Nottested	1,716 (47.4)	1,580 (41.2)	36 (57.1)	38 (55.9)	1,107 (42.4)	1,143 (38.1)	573 (60.5)	399 (51.5)
**HDL-C** ≥1.0 mmol/L								
Yes	1,713 (47.3)	1,590 (41.4)^**^	28 (44.4)	22 (32.3)^*^	1,340 (51.3)	1,278 (42.6)^**^	345 (36.4)	290 (37.4)^**^
No	240 (6.6)	768 (20.0)	1 (1.6)	9 (13.2)	201 (7.7)	654 (21.8)	38 (4.0)	105 (13.6)
Nottested	1,667 (46.1)	1,482 (38.6)	34 (54.0)	37 (54.4)	1,069 (41.0)	1,065 (35.5)	564 (59.6)	380 (49.0)
**Triglycerides** <2.0 mmol/L								
Yes	1,178 (32.5)	1,414 (36.8)^**^	27 (42.9)	22 (32.3)	902 (34.6)	1,141 (38.1)^**^	249 (26.3)	251 (32.4)^**^
No	902 (24.9)	1,071 (27.9)	4 (6.3)	11 (16.2)	748 (28.7)	891 (29.7)	150 (15.8)	169 (21.8)
Nottested	1,540 (42.5)	1,355 (35.3)	32 (50.8)	35 (51.5)	960 (36.8)	965 (32.2)	548 (57.9)	355 (45.8)
**Non-HDL-C** <2.5 mmol/L								
Yes	74 (2.0)	119 (3.1)^*^	0 (0.0)	2 (2.9)	61 (2.3)	102 (3.4)^*^	13 (1.4)	15 (1.9)
No	281 (7.8)	308 (8.0)	3 (4.8)	2 (2.9)	204 (7.8)	245 (8.2)	74 (7.8)	61 (7.9)
Not tested	3,265 (90.2)	3,413 (88.9)	60 (95.2)	64 (94.1)	2,345 (89.8)	2,650 (88.4)	860 (90.8)	699 (90.2)
**Urine albumin-creatinine ratio (uACR)**^b^								
Yes	1,062 (29.3)	1,144 (29.8)^**^	19 (30.2)	21 (30.9)	894 (34.2)	971 (32.4)^**^	149 (15.7)	152 (19.6)^**^
No	433 (12.0)	761 (19.8)	7 (11.1)	7 (10.3)	376 (14.4)	649 (21.6)	50 (5.3)	105 (13.5)
Nottested	2,125 (58.7)	1,935 (50.4)	37 (58.7)	40 (58.8)	1,340 (51.3)	1,377 (45.9)	748 (79.0)	518 (66.8)
**Glucose lowering medications** (ever prescription)^I^	2,884 (79.7)	3,091 (80.5)	60 (95.2)	64 (94.1)	2,163 (82.9)	2,511 (83.8)	661 (69.8)	516 (66.6)
**Glucose lowering medications** (prescription over a period of 395 days up to last encounter)^I^	1,894 (52.3)	2,334 (60.8)^**^	43 (68.3)	46 (67.7)	1,565 (60.0)	1,979 (66.0)^**^	286 (30.2)	309 (39.9)^**^
**Lipid modifying agents** (ever prescription)^II^	2,474 (68.3)	2,866 (74.6)^**^	24 (38.1)	36 (52.9)	1,943 (74.4)	2,323 (77.5)^*^	507 (53.5)	507 (65.4)^**^
**Lipid modifying agents** (prescription over a period of 395 days up to last encounter)^II^	1,751 (48.4)	2,237 (58.3)^**^	15 (23.8)	24 (35.3)	1,427 (54.7)	1,841 (61.4)^**^	309 (32.6)	372 (48.0)^**^
**Blood pressure lowering agents** (ever prescription)^III^	3,269 (90.3)	3,414 (88.9)^*^	40 (63.5)	48 (70.6)	2,417 (92.6)	2,698 (90.0)^*^	812 (85.7)	668 (86.2)
**Blood pressure lowering agents** (prescription over a period of 395 days up to last encounter)^III^	2,352 (65.0)	2,676 (69.7)^**^	19 (30.2)	32 (47.1)^*^	1,821 (69.8)	2,151 (71.8)	512 (54.1)	493 (63.6)^**^
**BMI** (kg/m^2^)								
Underweight: <18.5	5 (0.1)	2 (0.1)^**^	0 (0.0)	1 (1.5)^*^	3 (0.1)	0 (0.0)^**^	2 (0.2)	1 (0.1)^**^
Normal weight: 18.5-24.9	211 (5.8)	144 (3.7)	20 (31.7)	8 (11.8)	122 (4.7)	105 (3.5)	69 (7.3)	31 (4.0)
Overweight: 25.0-29.9	661 (18.3)	904 (23.5)	11 (17.5)	22 (32.3)	481 (18.4)	721 (24.1)	169 (17.8)	161 (20.8)
Obese class I: 30.0-34.9	909 (25.1)	1,203 (31.3)	11 (17.5)	14 (20.6)	668 (25.6)	953 (31.8)	230 (24.3)	236 (30.4)
Obese class II: 35.0-39.9	680 (18.8)	692 (18.0)	3 (4.8)	8 (11.8)	517 (19.8)	551 (18.4)	160 (16.9)	133 (17.2)
Obese class III: ≥40.0	826 (22.8)	595 (15.5)	4 (6.3)	0 (0.0)	625 (24.0)	482 (16.1)	197 (20.8)	113 (14.6)
Notmeasured	328 (9.1)	300 (7.8)	14 (22.2)	15 (22.1)	194 (7.4)	185 (6.2)	120 (12.7)	100 (12.9)
**Smoking**								
Non-smoker	1,507 (41.6)	1,087 (28.3)^**^	27 (42.9)	18 (26.5)	1,158 (44.4)	873 (29.1)^**^	322 (34.0)	196 (25.3)^**^
Current	279 (7.7)	367 (9.6)	7 (11.1)	13 (19.1)	209 (8.0)	308 (10.3)	63 (6.7)	46 (5.9)
Past	1,577 (43.6)	2,105 (54.8)	24 (38.1)	28 (41.2)	1,086 (41.6)	1,631 (54.4)	467 (49.3)	446 (57.6)
Notrecorded	257 (7.1)	281 (7.3)	5 (7.9)	9 (13.2)	157 (6.0)	185 (6.2)	95 (10.0)	87 (11.2)
**Influenza vaccination**, ever	1,881(52.0)	1,904 (49.6)^*^	22 (34.9)	34 (50.0)	1,479 (56.7)	1,557 (52.0)^**^	380 (40.1)	313 (40.4)
**Influenza vaccination**, over a period of 395 days up to last encounter	871 (24.1)	883 (23.0)	10 (15.9)	14 (20.6)	682 (26.1)	725 (24.2)	179 (18.9)	144 (18.6)
**Pneumococcal vaccination**, ever	401 (11.1)	434 (11.3)	1 (1.6)	4 (5.9)	333 (12.8)	378 (12.6)	67 (7.1)	52 (6.7)
**Ophthalmological review**, ever	1,289 (35.6)	1,384 (36.0)	21 (33.3)	23 (33.8)	1,076 (41.2)	1,186 (39.6)	192 (20.3)	175 (22.6)
**Referral to a podiatrist**, ever	630 (17.4)	698 (18.2)	11 (17.5)	10 (14.7)	477 (18.3)	574 (19.1)	142 (15.0)	114 (14.7)

*Abbreviations*: *BMI* body mass index, *BP* Blood pressure, *DM* Diabetes mellitus, *HbA1c* Glycated haemoglobin, *HDL* High density lipoprotein, *LDL* Low density lipoprotein

^a^LDL_C taret: <2.0 mmol/L or <1.8 mmol/L for those with established CVD (in this analysis these included coronary heart disease, cerebrovascular disease/stroke, or heart failure)

^b^Urine Albumin-creatine ratio (uARC): <3.5 mg/mmol in women and <2.5 mg/mmol in men

^I^ ATC code A10; ^II^ ATC code C10; ^III^ ATC codes C02-C04, C07-C09

The absence of pathology testing among individuals with either prevalent or incident diabetes was notably common, with a higher frequency observed in women compared to men, as indicated in Additional file 1 Table S4. After adjusting for age, BMI, smoking status, SEIFA-IRSD, Indigenous ethnicity, rurality, duration of follow-up, type of diabetes, and cluster effect, women were found to be 24% less likely than men to have their HbA1_c_ tested over the 395-day period leading up to the last clinical encounter. Similarly, women were 23% less likely to undergo cholesterol testing, 35% less likely to undergo kidney function screening, 17% less likely to have their blood pressure measured, and 42% less likely to receive treatment with a lipid-lowering agent (Additional file 1 Table S4).

However, compared to women, men smoked more and were less likely to achieve blood pressure and HbA_1c_ targets (Tables 3 and 4). The multivariable analysis that was limited to those with incident type 2 or unspecified diabetes who had at least three years of follow-up post diagnosis, found that men were 21% less likely than women to achieve the HbA1_c_ target (adjusted OR 0.79, 95% CI 0.69 – 0.91), *p* = 0.001. The area under the receiver operating characteristic curve of the model was 0.74 (95% CI 0.72 – 0.75) (Table 5).

**Table 5:** Multilevel mixed-effects logistic regression modelling “HbA1c ≤7.0% (≤53 mmol/mol)” over a period spanning 395 days up to the last clinical encounter in patients with incident diabetes (type 2 or unspecified diabetes) who had at least 3 years of follow-up post-diabetes

	Univariate		Multivariate^a^	
	OR (95% CI)	*P* value	OR (95% CI)	*P* value
**Men** (women as reference)	0.75 (0.67 – 0.85)	<0.001	0.79 (0.69 – 0.91)	0.001
**Age at last encounter** (years)				
18-49 (reference)	1.00		1.00	
50-59	1.27 (1.01 – 1.61)	0.041	1.27 (0.89 – 1.65)	0.069
60-69	1.67 (1.34 – 2.09)	<0.001	1.44 (1.12 – 1.86)	0.004
≥70	2.83 (2.29 – 3.50)	<0.001	2.01 (1.57 – 2.59)	<0.001
**BMI** (kg/m^2^)				
≤24.9 (reference)	1.00		1.00	
25.0 – 29.9	0.85 (0.59 – 1.22)	0.383	0.90 (0.61 – 1.32)	0.584
30.0 – 34.9	0.66 (0.47 – 0.94)	0.022	0.78 (0.53 – 1.14)	0.197
35.0 – 39.9	0.57 (0.40 – 0.82)	0.002	0.71 (0.48 – 1.05)	0.085
≥40	0.61 (0.43 – 0.87)	0.007	0.80 (0.54 – 1.19)	0.269
Unknown	0.55 (0.36 – 0.85)	0.007	0.72 (0.45 – 1.16)	0.179
**Smoking status**				
Non-smoker (reference)	1.00		1.00	
Past smoker	1.05 (0.91 – 1.22)	0.487	1.14 (0.97 – 1.34)	0.101
Smoker	0.62 (0.50 – 0.78)	<0.001	0.92 (0.72 – 1.18)	0.521
Unknown	0.78 (0.57 – 1.05)	0.104	0.83 (0.59 – 1.16)	0.269
**SEIFA-IRSD quintiles**				
1st (Lowest) (Reference)	1.00		1:00	
2nd	0.89 (0.64 – 1.23)	0.480	0.85 (0.60 – 1.19)	0.346
3rd	0.83 (0.60 – 1.14)	0.249	0.80 (0.57 – 1.12)	0.192
4th	0.94 (0.68 – 1.30)	0.702	0.93 (0.66 – 1.31)	0.679
5th (Highest)	0.98 (0.69 – 1.40)	0.928	0.96 (0.66 – 1.39)	0.831
Unknown	1.05 (0.46 – 2.43)	0.904	0.88 (0.36 – 2.16)	0.782
**Active status**^b^				
Active (reference)	1.00		1.00	
Inactive	1.01 (0.87 – 1.18)	0.870	1.06 (0.89 – 1.26)	0.535
Deceased	1.51 (1.19 – 1.93)	0.001	1.31 (1.01 – 1.71)	0.045
**Years of follow-up**, continuous	0.99 (0.98 – 1.01)	0.698	0.98 (0.97 – 0.99)	0.033
**Anaemia**^c^**, **yes	1.35 (1.11 – 1.64)	0.002	1.29 (1.04 – 1.59)	0.021
**Chronic liver disease, **yes	1.04 (0.63 – 1.73)	0.867	0.98 (0.57 – 1.69)	0.941
**Chronic kidney disease, **yes	1.44 (1.10 – 1.88)	0.008	1.25 (0.93 – 1.68)	0.143
**Hypertriglyceridaemia**^d^**, **yes	0.99 (0.72 – 1.37)	0.961	1.14 (0.81 – 1.62)	0.447
**Pregnancy**^e^, yes	0.96 (0.08 – 10.7)	0.971	0.60 (0.05 – 6.45)	0.641
**HbA1c baseline ever first recorded level,** continuous	0.57 (0.54 – 0.60)	<0.001	0.59 (0.56 – 0.63)	<0.001
**Receiver Operating Characteristic (ROC) curve (95% CI**)			0.74 (0.72 – 0.75)	

*Abbreviations*: *BMI* Body mass index, *HbA1c* Glycated haemoglobin, *SEIFA-IRSD* Socio-Economic Indexes for Areas – Index of Relative Socio-Economic Disadvantage

^a^The multivariate model was also adjusted for Indigenous status and intracluster correlations within the participating 39 general practices

^b^At the time of data extraction

^c^Anaemia, chronic or acute over a period spanning 395 days up to the last clinical encounter

^d^As coded by MedicineInsight, a yes/no variable

^e^Pregnancy over a period spanning 395 days up to the last clinical encounter

Similar results were found when, in sensitivity analyses, pregnant women were excluded from the model and/or when the model only included patients with type 2 diabetes.

## Discussion

This large population-based retrospective study that used routinely collected primary healthcare data validates the overall higher prevalence and incidence of diabetes in men as opposed to women. While discernible sex differences favouring men were observed in diabetes management, women were more likely to achieve blood pressure and HbA_1c_ targets. In contrast, women exhibiting a higher likelihood of obesity were less successful than men to meet blood lipid targets and were also less likely to receive treatment with a lipid lowering or blood pressure lowering or glucose lowering agent. This study highlights a substantially higher prevalence of diabetes-related conditions and comorbidities in men compared to women, including elevated rates of retinopathy, nephropathy, neuropathy, coronary heart disease, and heart failure.

Similar to other studies, we report an overall higher prevalence of diabetes in men compared to women [36], a higher incidence rate in young women (aged ≤ 30 years) [37] but higher incidence rates in men in older patients [38]. In this large sample of Australian adults with a record-based diagnosis of diabetes, there is evidence of sex differences in diabetes incidence diagnosis, with trends increasing in men as they aged. The higher risk of being diagnosed with diabetes in men was not explained by age, BMI, smoking status, socioeconomic status, and years of follow-up. In our sample, women with diabetes were more likely than men to be living with morbid obesity. The information we had on waist circumference was incomplete, precluding its use in the analysis. An explanation for the observed higher risk of diabetes in men compared to women may relate to sex differences in body fat storage. Subcutaneous and lower extremity fat storage is more common in women, while men tend to store fat in the abdominal region. Consequently, men exhibit significantly higher levels of visceral and ectopic fat than premenopausal women, irrespective of BMI and total body fat. The selective accumulation of excess fat in visceral and ectopic tissues in men may accelerate the onset of insulin resistance and diabetes [39]. In contrast, women might need to accumulate more weight, and their metabolic risk factors may need to deteriorate to a greater extent than in men to attain the same levels of visceral and ectopic fat necessary for developing insulin resistance and eventual diabetes [40]. Postmenopausal women tend to store more abdominal visceral fat, similar to patterns typically seen in men [41].

Studies on sex differences in quality-of-care indicators and in diabetes management are inconclusive [42–44]. The National Diabetes Audit, evaluating essential care processes and treatment target attainment in individuals living with diabetes reported that women were less inclined than men to receive screening of risk factors and risk factors control, with women being less likely than men to undergo risk factor assessments for smoking status, BMI, foot surveillance, cholesterol levels, and urine albumin. However, women were more prone to undergo testing for serum creatinine and blood pressure [42]. A large population-based study conducted in Italy, involving 415,294 individuals with type 2 diabetes, indicated that women were less likely to receive recommended care compared to men. Specifically, women were less likely to undergo assessments for kidney function, ophthalmological review, and foot surveillance, with women, who were more likely to have a BMI ≥ 30 kg/m^2^ than men, facing more challenges in achieving risk factor control for HbA_1c_ and LDL-cholesterol despite drug intervention and were less likely to receive adequate treatment in the presence of micro/macroalbuminuria compared to men [43]. In contrast, a cross-sectional study involving 17,702 individuals with diabetes in the United States, drawn from the Medical Expenditure Panel Survey Household Component, showed that women were more inclined to receive recommended care compared to men over a nine-year study duration [44]. In adjusted analyses, women demonstrated a higher likelihood of undergoing annual tests for dilated eye exams and blood pressure control, as well as visiting a doctor. No disparities were observed in HbA_1c_ testing and foot surveillance compared to men [44].

The RACGP advises to frequently assess HbA1_c_ levels in patients with established diabetes. The HbA1_c_ test is listed on the MBS for subsidy once every 12 months for the diagnosis of diabetes in high-risk individuals, and up to four times per year for monitoring of established diabetes [27]. In our study, overall, 14,843 out of 34,551 individuals (42.9%) did not undergo an HbA1_c_ test over a period spanning 395 days up to their last clinical encounter. The percentages of non-adherence to recommended tests were consistently higher in women compared to men, indicating suboptimal management of established diabetes. This disparity extended beyond HbA1_c_ testing, affecting women's access to essential screenings such as lipid levels, urine-albumin creatine tests, and blood pressure measurements. Additionally, our findings show that women were significantly less likely than men to receive treatment with a lipid lowering or blood pressure lowering or glucose lowering agent.

Men compared to women had more comorbidities and diabetes-associated conditions. Number of consultations did not vary by sex; however, we had no information on compliance with treatment and whether this differed by sex. Non-compliance with long-term medication for conditions like diabetes, hypertension, and dyslipidaemia is not uncommon, leading to compromised health risks [45]. Nonetheless, the reported association of sex/gender with compliance to long-term diabetes medications has not been consistent. Male sex has shown a positive association with compliance [46], a negative association with compliance [47], and no association with compliance [48, 49]. Gender differences in the perception and self-management of the disease have been also reported. Women often take their disease more seriously, reporting a higher impact on their daily life and are more involved in self-management than men [50].

### Strengths and limitations

Strengths of this study include its population-based provenance, the longitudinal design, the routinely collected primary healthcare data, and the study’s broaden generalisability. Similarly, our inclusion of all patients irrespective of level of engagement with the health services has made the sample more representative of the wider primary care population. However, the study has limitations. Although MedicineInsight's coverage in Western Australia represents the general population of practices [51], the 39 participating practices in our study may not fully represent all clinics. We had no information on compliance and dispensing data, nor on individuals who may have moved to other general practices where their treatment was resumed. Misclassification of diabetes type could have occurred as adult-onset insulin-dependent diabetes that did not specifically categorise patients as having type 2 diabetes was classified as type 1. The research might have underestimated the percentage of patients undergoing optimal treatment, especially if patients received care in alternative settings (such as different general practices or hospitals), or if the patient's present medication record was incomplete, or if the patient records were not updated at the time of data extraction. While we used reason for consultation, we had no access to the full consultation notes. The multivariable analysis that investigated glycaemic control included only those with a known HbA1_c_ level. Multiple imputations to complete the missingness in HbA1_c_ levels was out of the scope of this study. The aim of this study was not to investigate initial management of diabetes upon diagnosis or change in HbA1c levels over time. The conditions described as “ever recorded” were extracted from any entries in the GP records, both before and after the diabetes diagnosis, as such that these conditions did not necessarily indicate complications that developed post-diabetes. Lastly, we had no information whether women with diabetes and confirmed dyslipidaemia were less treated with lipid modifying agents due to intolerance to statins.

## Conclusions

This study used routinely collected primary healthcare data to show sex disparities in the management of diabetes in Australia. Compared to men, women with diabetes were less likely to undergo lipid and kidney function screening but were more likely than men to achieve blood pressure and HbA_1c_ health targets. Men were significantly more likely than women to have retinopathy, nephropathy, neuropathy, coronary heart disease, heart failure, peripheral vascular disease, and peripheral artery disease. Our findings indicate that diabetes management should take into account the sex of the patient.

## Supplementary Information

Additional file 1: Tables S1-S4. Table S1. Characteristics of study sample by ever recorded diabetes mellitus status. Table S2. Risk of being diagnosed with diabetes (incident cases): Weibull regression. Table S3. Ever recorded conditions by sex at last clinical encounter, all types of diabetes combined: n (%). Table S4. Adjusted odds ratio of not having a test or measure assessed and not being managed with medications over a period of 395 days up to the last clinical encounter (if not otherwise stated) in individuals with diabetes (all types combined): comparing women to men.

## Data Availability

All data generated or analysed during this study are included in this published article (and its Additional file 1).

## Abbreviations

BMI: Body mass index
BP: Blood pressure
CHD: Coronary heart disease
DKA: Diabetic ketoacidosis
DM: Diabetes mellitus
GP: General practitioner
HbA1c: Glycated haemoglobin
HDL: High density lipoprotein
HONK: Hyperglycaemic non-ketotic coma
IQR: Interquartile range
LDL: Low-density lipoprotein
MBS: Medicare Benefits Schedule
PAD: Peripheral artery disease
PVD: Peripheral vascular disease
SD: Standard deviation
SEIFA-IRSD: Socio-Economic Indexes for Areas – Index of Relative Socio-Economic Disadvantage
TIA: Transient ischaemic attack
uACR: Urine albumin-creatinine ratio
