# Supplementary material for: Sex disparities in the prevalence, incidence, and management of diabetes mellitus: an Australian retrospective primary healthcare study involving 668,891 individuals
Source: BMC Med. 2024 Oct 16;22:475. doi: 10.1186/s12916-024-03698-0 (PMC11483993; doi:10.1186/s12916-024-03698-0)
Supplement: Supplementary file 1 — Additional file 1: Tables S1-S4. Table S1. Characteristics of study sample by ever recorded diabetes mellitus status. Table S2. Risk of being diagnosed with diabetes (incident cases): Weibull regression. Table S3. Ever recorded conditions by sex at last clinical encounter, all types of diabetes combined: n (%). Table S4. Adjusted odds ratio of not having a test or measure assessed and not being managed with medications over a period of 395 days up to the last clinical encounter (if not otherwise stated) in individuals with diabetes (all types combined): comparing women to men. [file 12916_2024_3698_MOESM1_ESM.docx]

**Additional File 1**

**Table S1.** Characteristics of study sample by ever recorded diabetes mellitus status.

**Table S2.** Risk of being diagnosed with diabetes (incident cases): Weibull regression.

**Table S3.** Ever recorded conditions by sex at last clinical encounter, all types of diabetes combined: n (%).

**Table S4.** Adjusted odds ratio of not having a test or measure assessed and not being managed with medications over a period of 395 days up to the last clinical encounter (if not otherwise stated) in individuals with diabetes (all types combined): comparing women to men.

**Table S1** Characteristics^!^ of study sample by ever recorded diabetes mellitus status.

| **Characteristics** | **All**  **N=668,891**  **(100.0%)** | **Without diabetes**  **N=634,232**  **(94.8%)** | **With diabetes**  **N=34,659**  **(5.2%)** |
| --- | --- | --- | --- |
| **Age at first adult clinical encounter (years),** mean (SD) | 40.5 (17.1) | 39.7 (16.8) | 54.4 (16.0) |
| **Age at last clinical encounter (years),** mean (SD) | 44.2 (18.1) | 43.2 (17.7) | 61.7 (16.6) |
| **Follow-up (years),** mean (SD), median [IQR] | 3.7 (5.1), 1.4 [0.02, 5.5] | 3.5 (5.0), 1.2 [0.01, 5.2] | 7.3 (6.5), 5.5 [1.7, 11.5] |
| **Sex**, n (%)  Men  Women  Unknown | 305,719 (45.7)  356,910 (53.4)  6,262 (0.9) | 288,133 (45.4)  339,945 (53.6)  6,154 (1.0) | 17,586 (50.7)  16,965 (48.9)  108 (0.3) |
| **Index of relative socio-economic advantage and disadvantage quintiles**  1^st^ (Lowest)  2^nd^  3^rd^  4^th^  5^th^ (Highest) (reference)  Unknown | 42,975 (6.4)  156,011 (23.3)  172,030 (25.7)  171,195 (25.6)  119,737 (17.9)  6,943 (1.0) | 40,515 (6.4)  146,420 (23.1)  162,198 (25.6)  163,761 (25.8)  114, 673 (18.1)  6,665 (1.0) | 2,460 (7.1)  9,591 (27.7)  9,832 (28.4)  7,434 (21.4)  5,064 (14.6)  278 (0.8) |
| **Residence regionality**, n (%)  Major cities  Inner regional  Outer regional  Remote or very remote  Unknown | 482,493 (72.1)  104,913 (15.7)  32,862 (4.9)  41,680 (6.2)  6,943 (1.0) | 457,745 (72.2)  100,158 (15.8)  30,438 (4.8)  39,226 (6.2)  6,665 (1.0) | 24,748 (71.4)  4,755 (13.7)  2,424 (7.0)  2,454 (7.1)  278 (0.8) |
| **Body mass index over a period of five years from the first adult clinical encounter**, n (%)  Underweight: <18.5 kg/m^2^  Normal weight: 18.5-24.9 kg/m^2^  Overweight: 25.0-29.9 kg/m^2^  Obese class I: 30.0-34.9 kg/m^2^  Obese class II: 35.0-39.9 kg/m^2^  Obese class III: ≥40.0 kg/m^2^  Not measured | 4,039 (0.6)  63,924 (9.6)  72,549 (10.8)  46,476 (6.9)  20,954 (3.1)  14,404 (2.1)  446,545 (66.8) | 3,982 (0.6)  61,843 (9.7)  66,984 (10.6)  40,285 (6.3)  17,010 (2.7)  11,069 (1.7)  433,059 (68.3) | 57 (0.2)  2,081 (6.0)  5,565 (16.1)  6,191 (17.9)  3,944 (11.4)  3,335 (9.6)  13,486 (38.9) |
| **Number of consultations over a period of 395 days up to the last clinical encounter**, mean (SD), median [IQR] | 3.8 (5.3), 2 [1, 4] | 3.6 (4.9), 2 [1, 4] | 7.2 (9.5), 3 [1, 9] |
| **Patient activity^!!^ status at data extraction**, n (%)  Active  Inactive  Deceased | 202,026 (30.2)  458,113 (68.5)  8,752 (1.3) | 184,936 (29.2)  442,746 (69.8)  6,550 (1.0) | 17,090 (49.3)  15,367 (44.3)  2,202 (6.3) |

^!^ All characteristics of those with and without diabetes were statistically different at a p <0.001.

^!!^Active patients were defined as those who had had at least three encounters for any reason with a general practitioner (GP) during the two years prior to data extraction (January 2022).

**Table S2**: Risk of being diagnosed with diabetes (incident cases): Weibull regression^!^

| **Covariates** | **Hazard ratio (95% CI)** | **P value** |
| --- | --- | --- |
| **Age at first adult clinical encounter**, continuous | 0.97 (0.96 – 0.98) | <0.001 |
| **Male**, (female as reference) | 1.19 (1.09 – 1.30) | <0.001 |
| **Smoking status at first adult clinical encounter**  Non-smoker (reference)  Past smoker  Current smoker  Unknown status | 1.00  1.12 (1.01 – 1.25)  1.17 (1.07 – 1.27)  0.86 (0.74 – 1.01) | 0.035  <0.001  0.066 |
| **Index of relative socio-economic advantage and disadvantage quintiles**  1^st^ (Lowest)  2^nd^  3^rd^  4^th^  5^th^ (Highest) (reference)  Unknown | 1.06 (0.85 – 1.33)  1.26 (1.03 – 1.53)  1.07 (0.93 – 1.24)  1.26 (1.07 – 1.49)  1.00  1.13 (0.87 – 1.48) | 0.591  0.021  0.336  0.006  0.353 |
| **BMI at first adult clinical encounter** (kg/m^2^)  ≤24.9 (reference)  25.0 – 29.9  30.0 – 34.9  ≥35.0  Unknown | 1.00  2.26 (1.97 – 2.60)  4.59 (3.84 – 5.50)  9.09 (7.76 – 10.65)  1.56 (1.27 – 1.91) | <0.001  <0.001  <0.001  <0.001 |

^!^ Also adjusted for Indigenous ethnicity and cluster effect within the 39 general practices

**Table S3**: Ever recorded conditions by sex at last clinical encounter, all types of diabetes combined: n (%)^!^

|  | **Women**  **N=16,965** | **Men**  **N=17,586** | **P value** |
| --- | --- | --- | --- |
| **Retinopathy** | 368 (2.2) | 498 (2.8) | <0.001 |
| **Nephropathy** | 1,149 (6.8) | 1,404 (8.0) | <0.001 |
| **Neuropathy** | 1,245 (7.3) | 1,453 (8.3) | 0.001 |
| **Hypertension** | 8,160 (48.1) | 9,023 (51.3) | <0.001 |
| **Dyslipidaemia** | 6,216 (36.6) | 6,782 (38.6) | <0.001 |
| **Peripheral Vascular Disease / Peripheral Artery Disease** | 290 (1.7) | 574 (3.3) | <0.001 |
| **Coronary Heart Disease** | 2,088 (12.3) | 3,470 (19.7) | <0.001 |
| **Heart Failure** | 782 (4.6) | 1,042 (5.9) | <0.001 |
| **Stroke / Transient Ischaemic Attack** | 1,658 (9.8) | 1,740 (9.9) | 0.705 |
| **Cancer** | 3,236 (19.1) | 3,435 (19.5) | 0.281 |
| **Metabolic^$^** | 148 (0.9) | 112 (0.6) | 0.011 |
| **Other^^^** | 93 (0.6) | 68 (0.4) | 0.028 |

! Not including 108 patients with unknown sex

^$^ Metabolic conditions included diabetic ketoacidosis (DKA), hyperglycaemic non-ketotic coma (HONK), and hypoglycaemia.

^ Other included cheiroarthropathy and periodontitis

**Table S4:** Adjusted odds ratio^!^ of not having a test or measure assessed and not being managed with medications over a period of 395 days up to the last clinical encounter (if not otherwise stated) in individuals with diabetes (all types combined): comparing women to men.

|  | **Prevalent^#^** | **Incident^##^** | **All** |
| --- | --- | --- | --- |
| **Test / measure / management item** | **Odds Ratio (95% CI), p value** | **Odds Ratio (95% CI), p value** | **Odds Ratio (95% CI), p value** |
| HbA1_c_ | 1.26 (1.15 – 1.38), p<0.001 | 1.40 (1.26 – 1.56), p<0.001 | 1.24 (1.18 – 1.30), p<0.001 |
| Blood pressure | 1.35 (1.22 – 1.49), p<0.001 | 1.24 (1.11 – 1.40), p<0.001 | 1.17 (1.11 – 1.23), p<0.001 |
| Total cholesterol | 1.24 (1.14 – 1.35), p<0.001 | 1.31 (1.19 – 1.45), p<0.001 | 1.23 (1.18 – 1.29), p<0.001 |
| LDL-C | 1.17 (1.07 – 1.28), p<0.001 | 1.25 (1.14 – 1.38), p<0.001 | 1.16 (1.10 – 1.21), p<0.001 |
| HDL-C | 1.21 (1.11 – 1.32), p<0.001 | 1.32 (1.20 – 1.46), p<0.001 | 1.23 (1.17 – 1.29), p<0.011 |
| Triglycerides | 1.24 (1.14 – 1.36), p<0.001 | 1.32 (1.20 – 1.46), p<0.001 | 1.24 (1.18 – 1.30), p<0.001 |
| Non-HDL-C | 1.16 (0.99 – 1.35), p=0.070 | 1.22 (1.04 – 1.43), p=0.017 | 1.14 (1.05 – 1.24), p=0.002 |
| Urine albumin-creatinine | 1.26 (1.15 – 1.37), p<0.001 | 1.38 (1.25 – 1.52), p<0.001 | 1.35 (1.29 – 1.42), p<0.001 |
| Management with glucose lowering medications | 1.30 (1.19 – 1.43), p<0.001 | 1.36 (1.23 – 1.50), p<0.001 | 1.13 (1.08 – 1.18), p<0.001 |
| Management with lipid modifying agents | 1.36 (1.25 – 1.49), p<0.001 | 1.44 (1.30 – 1.59), p<0.001 | 1.42 (1.35 – 1.49), p<0.001 |
| Management with blood pressure lowering agents | 1.18 (1.08 – 1.30), p<0.001 | 1.23 (1.10 – 1.37), p<0.001 | 1.12 (1.07 – 1.18), p<0.001 |
| Influenza vaccination | 0.94 (0.85 – 1.04), p=0.211 | 0.93 (0.83 – 1.04), p=0.236 | 0.94 (0.88 – 0.99), p=0.030 |
| Pneumococcal vaccination, ever | 1.03 (0.89 – 1.20), p=0.647 | 1.03 (0.87 – 1.20), p=0.752 | 1.05 (0.96 – 1.16), p=0.285 |
| Ophthalmological review | 0.92 (0.84 – 1.02), p=0.106 | 0.92 (0.83 – 1.03), p=0.135 | 0.94 (0.89 – 1.00), p=0.064 |
| Referral to a podiatrist | 0.98 (0.87 – 1.11), p=0.783 | 0.93 (0.82 – 1.07), p=0.314 | 0.95 (0.88 – 1.03), p=0.224 |

! Adjusted for age, BMI, smoking status (all three as measured at last encounter), SEIFA-IRSD, Indigenous ethnicity, rurality, duration of follow-up, type of diabetes, and cluster effect within the 39 participating general practices.

^#^ Prevalent cases with adult follow-up of ≥3 years.

^##^ Incident cases with adult follow-up of ≥3 years following diagnosis of diabetes.
